# Direct Hospitalization Cost of Patients with Acute Exacerbation of Chronic Obstructive Pulmonary Disease in Vietnam

**DOI:** 10.3390/ijerph16010088

**Published:** 2018-12-30

**Authors:** Chau Quy Ngo, Thuy Thi Bui, Giap Van Vu, Hanh Thi Chu, Phuong Thu Phan, Ha Ngoc Pham, Giang Thu Vu, Long Hoang Nguyen, Giang Hai Ha, Bach Xuan Tran, Carl A. Latkin, Cyrus S. H. Ho, Roger C. M. Ho

**Affiliations:** 1 Department of Internal Medicine, Hanoi Medical University, Hanoi 100000, Vietnam; ngoquychaubmh@gmail.com (C.Q.N.); buithuyhmu@gmail.com (T.T.B.); vuvangiap@hmu.edu.vn (G.V.V.); thuphuongdr@gmail.com (P.T.P.); 2 Respiratory Center, Bach Mai Hospital, Hanoi 100000, Vietnam; chuthihanhbmh@gmail.com (H.T.C.); drhaphamngoc@gmail.com (H.N.P.); 3 Center of Excellence in Evidence-Based Medicine, Nguyen Tat Thanh University, Ho Chi Minh City 70000, Vietnam; giang.coentt@gmai.com; 4 Center of Excellence in Behavioral Medicine, Nguyen Tat Thanh University, Ho Chi Minh City 70000, Vietnam; longnh.ph@gmail.com (L.H.N.); pcmrhcm@nus.edu.sg (R.C.M.H.); 5 Institute for Global Health Innovations, Duy Tan University, Da Nang 55000, Vietnam; giang.ighi@gmail.com; 6 Institute for Preventive Medicine and Public Health, Hanoi Medical University, Hanoi 100000, Vietnam; 7 Johns Hopkins Bloomberg School of Public Health, Baltimore, MD 21205, USA; carl.latkin@jhu.edu; 8 Department of Psychological Medicine, National University Hospital, Singapore 119074, Singapore; cyrushosh@gmail.com; 9 Department of Psychological Medicine, Yong Loo Lin School of Medicine, National University of Singapore, Singapore 119077, Singapore

**Keywords:** acute exacerbation, co-morbidity, COPD, cost, economics, hospitalization

## Abstract

Acute exacerbations of chronic obstructive pulmonary disease (AECOPD) have been found to contribute, predominantly, to increasing costs of COPD—a major public health issue. This study aimed to fill the gap in literature concerning costs of AECOPD in Vietnam, by examining the direct cost of AECOPD hospitalization and determining potentially associated factors. A cross-sectional study was conducted at the Respiratory Center of Bach Mai Hospital, Hanoi. A total of 57 participants were selected. Information regarding sociodemographic features, clinical characteristics, and hospitalization costs were collected. A multivariate generalized linear regression model was utilized to determine the factors associated with hospitalization costs. The mean total and daily hospitalization cost were 18.3 million VND (SD = 12.9) and 2.5 million VND (SD = 3.2), respectively. Medication cost accounted for 53.9% of hospitalization cost (from 44.0% in the Global Initiative for Chronic Obstructive Lung Disease Classification A (GOLD A) to 55.3% in GOLD C). Patients having GOLD D COPD (Coef. = 5.78; 95% CI = 0.73–10.83), higher age (Coef. = 0.37; 95% CI = 0.13–0.61), and higher duration of hospitalization (Coef. = 1.91; 95% CI = 1.28–2.53) had higher hospitalization costs (*p* < 0.05). This study suggested that interventions to screen COPD patients as well as provide timely treatment should be conducted widely in the community in order to avoid any unnecessary hospitalization cost, consequently reducing the economic burden of COPD.

## 1. Introduction

Chronic obstructive pulmonary disease (COPD) has been considered a growing threat to global health in the 21st century, as the world population ages, smoking rates increase, and air pollution becomes more severe, especially in Asian regions [1]. The Global Burden of Disease Study reported an estimated 251 million cases of COPD globally in 2016 [2], which was 3.86 times the number of cases estimated in 2005 [3]. COPD was estimated to result in 3.2 million fatal cases worldwide [4], making it the third leading cause of death and, expectedly, the number one fatality cause by 2030 [5]. Low- and middle-income countries (LMICs) have been hardest hit in terms of COPD fatality, with over 90% of deaths occurring there [2]. The contraction of COPD also affects individuals and the society through the disability it involved—in 2015 COPD was responsible for 2.6% of global Disability-Adjusted Life Years, taking the eighth rank among 315 global burden of disease causes [4].

Increasing prevalence and severity of impacts of COPD would intensify the economic burden of the disease on the infected and on the society, more so in resource-poor regions [6]. Experts in the field have projected the economic impact of COPD in LMICs to reach £1.7 trillion by 2030 [5]. Acute exacerbations of COPD (AECOPD) have been found by a number of studies to contribute, predominantly, to costs of COPD, due to the hospitalized episodes such conditions induced [7,8,9,10]. Understanding the components of the hospitalization cost of AECOPD, as well as its potential influencing factors, thus, would be critical in ensuring efficient use of resources in AECOPD treatment and reducing total financial burden caused by the diseases [11].

There have been a few attempts in literature to determine the prevalence of COPD in Vietnam. A study using a prevalence model to estimate burden of COPD in 12 Asian countries, reported a COPD prevalence rate of 6.7% in Vietnam [12]. A more recent one conducted on patients in the north of Vietnam indicated the result of 7.1% prevalence [13]. Exacerbations were found to occur in over half of COPD patients [14], resulting in them having to attend hospitals. To our knowledge, there has yet to be a study on hospitalization costs of AECOPD in Vietnamese patients, though total cost of COPD would expectedly be substantial, compared to other smoking-related diseases. In 2005, health expenditure on COPD in Vietnam was estimated to be US$68.9 million, and accounted for 88.9% of the total cost of smoking-related health care, which was equivalent to 0.22% of the gross domestic product of Vietnam [15]. This study aimed to examine the direct cost of AECOPD hospitalization and determine potentially associated factors, consequently to identify areas for possible improvements in cost reduction, resources utilization, and general disease management.

## 2. Methods

### 2.1. Study Design and Setting

A cross-sectional study was conducted at the Respiratory Center of Bach Mai Hospital, Hanoi, from August to September 2018. This center is the leading facility in the North Vietnam for diagnosis and treatment of respiratory-related conditions, thus having a diverse patient population of various severity levels and socioeconomic backgrounds, of which many were transferred from health facilities at lower administrative levels. There are 20 to 30 patients with AECOPD being hospitalized to the Center each month.

### 2.2. Participants

Eligibility criteria for participating in the study were: (1) being at least 18 years old; (2) diagnosed with AECOPD following criteria of the Global Initiative for Chronic Obstructive Lung Disease (GOLD) 2017 [16]; (3) being treated at the Center as an inpatient; and (4) agreeing to be involved in the study by giving written consent. We excluded candidates that were pregnant at the time of study. A total of 57 participants were enrolled.

### 2.3. Measurements and Instruments

Records of recruited participants were withdrawn from the archive system of the Center. Information regarding sociodemographic features, clinical characteristics, and hospitalization costs were collected, details as below.

#### 2.3.1. Sociodemographic Features

Data about socio-demographic characteristics were collected including age, gender, living location, and smoking history of participants.

#### 2.3.2. Clinical Characteristics

Data regarding disease stage (GOLD A, B, C, D), comorbidities, days of being hospitalized, and number of times hospitalized due to AECOPD in the last year were collected via medical records.

#### 2.3.3. Hospitalization Costs

Costs of bed-day, diagnostic and imaging tests, procedures, medication, equipment, and supplies were collected from the finance and accounting system of the Bach Mai Hospital. This system contained all the inpatient costs before being covered by the health insurance.

Medication cost was calculated by summing total cost for antibiotic, bronchodilator, corticosteroid, and infusion solution, and other medications that patients received during hospitalization. Meanwhile, costs for equipment aids, oxygen therapy, and other medical supplies were classified into “equipment and supplies” cost components. Procedure-related costs included spirometry measures, and minor or major surgeries that were required during treatment.

### 2.4. Statistical Analysis

Stata version 15 was used to analyze the data. Kruska–Wallis test was adopted to examine the differences of sociodemographic, clinical, and cost characteristics between different disease stages. A multivariate generalized linear regression model, with Gaussian family and identity-link, was utilized to identify factors associated with the COPD hospitalization cost. Statistical significance was acknowledged at *p*-value of less than 0.05. 

### 2.5. Ethical Approval

The protocol of the study was reviewed and approved by The Institutional Review Board of Vietnam Respiratory Society (No:04/QD-VNRS).

## 3. Results

Of 57 patients, the majority of them were male (93.0%) and living in rural area (61.4%). The mean age of patients was 67.8 (SD = 8.0) years old. There were 15.8% of patients currently smoking. About comorbidities, the rates of hypertension, diabetesmellitus, congestive heart failure, coronary artery diseases, corpulmonale, and gastroesophageal reflux disease (GERD) were 19.3%, 12.3%, 15.8%, 5.3%, 22.8%, and 7.0%, respectively. The proportion of patients using antibiotic, bronchodilator, corticosteroid, and infusion solution were 96.5%, 100.0%, 100.0%, 93.0%, and 96.5%, respectively (Table 1).

The mean total cost of patients with acute exacerbation of COPD is shown in Table 2. Overall, the total cost was 18.3 million VND (SD = 12.9), and the hospitalization cost per day was 2.5 mil VND (SD = 3.2). The mean total cost varied from GOLD A with 6.3 mil VND (SD = 0.4) to GOLD D with 20.9 mil VND (SD = 14.9). The difference among patients with different stages of disease was statistically significant (*p* < 0.05).

**Figure 1:**
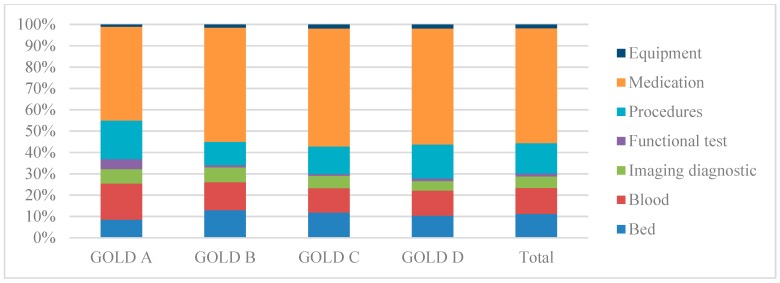
Distribution of cost components according to disease stages.

Figure 1 reveals the distribution of cost components according to disease stages. Medication cost accounted for the highest proportion of hospitalization cost with 53.9%, ranging from 44.0% in GOLD A to 55.3% in GOLD C. Meanwhile, functional test cost had the lowest percentage with 1.3%.

In the multivariate regression model, patients having GOLD D COPD (Coef. = 5.78; 95% CI = 0.73–10.83), higher age (Coef. = 0.37; 95% CI = 0.13–0.61), and higher duration of hospitalization (Coef. = 1.91; 95% CI = 1.28–2.53) had a higher hospitalization cost (*p* < 0.05) (Table 3).

## 4. Discussion

This study provides an insight into the hospitalization cost of COPD patients with acute exacerbation in the tertiary care setting in Vietnam. In this study, we estimated that the mean cost of this patient group was 18.3 million VND (~US$795.7). We also found that after adjustment, disease stages, age, and duration of hospitalization were correlated to the hospitalization cost, implying several clinical implications to reduce the hospitalization cost.

The finding of this study showed that the mean cost for treatment of AECOPD was 18.3 million VND and hospitalization cost per day was 2.1 million VND. This result was nearly four times higher than the mean cost per day of treatment in the study of Vu et. al in Vietnam (0.507 million VND/day) [17]. The reason for this might be due to the difference in prices of medication, supplies at each year, and different service techniques fee. The hospitalization cost in this study was quite similar to another study in Vietnam (2011), which showed that the hospitalization cost for a patient hospitalized with AECOPD was 18.2 million VND [18]. Our result was a bit lower than a study in Turkey (2012), where the mean cost of hospitalization was $889 (about 20.5 million VND with the 2012 exchange rate) [19]. This figure was higher than the cost of COPD in a State Hospital Chest Diseases Clinic in 2012 at $614 (about 12.7 million VND, 2012 exchange rate) [20]. The differences between health systems and rates in different hospitals and countries led to difficulty in direct comparison between our results and findings in other countries. However, collectively, the hospitalization cost for AECOPD might cause economic burden for patients and their families, as well as for the society, requiring good disease control and management, as well as prevention of acute exacerbation on patients to reduce the hospitalization cost.

Regarding distribution of cost components, the highest cost was for medication (53.9% of total hospitalization cost) and the lowest was for functional test (1.3%). Our research result was similar to Phan (2012) in Hanoi, which showed that the medication cost for AECOPD accounted for the highest proportion (69.6% of the total cost), followed by te procedures cost (16.9%) [18]. Another study of Vu et al. showed that the spending on medications, blood, and infusion was the highest, accounting for 75%; followed by preclinic cost (20%), bed cost (2%), consumable materials cost (2%), and procedures cost (1%) [17]. This was similar to a multi-centered study performed in China, which estimated that the percentage of medication cost to total cost was the highest (71.2%), followed by the procedures cost (16.7%) [21]. Our figure was different from a study in Turkey in 2012, which revealed that the medication cost accounted for the highest proportion (35%), followed by the bed cost (25.9%) [20].

Our result found that the disease stage was the significant associated factor with the hospitalization cost among patients with AECOPD. This finding was in line with other studies. In the United States, the mean cost in outpatients, patients hospitalized, patients with co-morbidities, and patients with intensive care and/or endotracheal intubation was $US647, $US7242, $US20,757, and $US44,909, respectively [22]. Meanwhile, in Taiwan, the mean cost of patients with Level A (FEV1 50%–80%) was 38,203 Taiwan dollars; 149,031 Taiwan dollars for Level B patients and 288,825 Taiwan dollars for Level C patients [23]. In Sweden, the estimated cost for patients with a mild level of COPD was $US18.36, while those having moderate, severe, and very severe level were $54.16, $322.98, and $33,4337, respectively [24]. These studies suggested that the hospitalization cost increased with the increase of severity of COPD. Therefore, timely screening and early treatment should be provided to the patients in order to avoid the progression of the disease, which could help to reduce the cost of hospitalization [24,25].

In this study, we also found that higher duration of hospitalization was related to higher hospitalization cost, which was similar to other studies in the world. For example, a study in Turkey found that the number of co-morbidities, number of hospitalizations in the past year, and the duration of hospitalization were among the factors that increased the cost significantly [19]. The result of a study in China (2008) found that cost positively corelated to hospitalization days and the number of hospitalization co-morbidities [21]. However, in the current study, our result showed no significant relationship between comorbidities and the direct hospitalization cost of acute exacerbation COPD. This finding was similar to the study in Turkey (2012) [19].

This study has several limitations. First, our study had a small sample size and used a convenient sampling technique, resulting in the constrained generalizability of the study results. Second, we employed a retrospective study design, which largely depended on the quality of existing data. We addressed this issue by standardizing the instrument, as well as calling the patients to verify the information in the medical records. Third, this study was performed in only one tertiary hospital, which might limit our generalizability to other hospital settings. Thus, further studies with a larger sample size in more hospitals would be warranted.

## 5. Conclusions

In conclusion, this study underlined the significantly high cost of hospitalization among patients with acute exacerbation COPD, especially in patients with higher age, higher duration of treatment, and higher level of severity. This study suggested that interventions to screen COPD patients should be conducted widely in the community in order to avoid any unnecessary cost of hospitalization, which could reduce the economic burden due to COPD.

## Figures and Tables

**Table 1:** Demographic and clinical characteristics (*n* = 57).

Characteristics	*n*	%
Gender, male	53	93.0
Living location, rural	35	61.4
Current smoker	9	15.8
Comorbidities		
Hypertension	11	19.3
Diabetes mellitus	7	12.3
Congestive heart failure	9	15.8
Coronary artery diseases	3	5.3
Cor pulmonale	13	22.8
Gastroesophageal reflux disease (GERD)	4	7.0
Medications		
Antibiotic	55	96.5
Bronchodilator	57	100.0
Corticosteroid	57	100.0
Infusion solution	53	93.0
Others	55	96.5
	Mean	SD
Age	67.8	8.0
Number of times hospitalized due to AECOPD in the last 12 months	1.6	2.0

**Table 2:** Cost categories according to disease stages (*n* = 57). Unit: Million VND.

Characteristics	Disease Stages										*p*-Value
	GOLD A		GOLD B		GOLD C		GOLD D		Total		
	Mean	SD	Mean	SD	Mean	SD	Mean	SD	Mean	SD	
Hospitalization cost											
Bed-day	0.5	0.2	1.5	0.5	2.2	2	1.9	1.3	1.8	1.4	0.07
Blood tests	1.1	0.7	1.7	1.2	1.8	0.8	2.1	1.2	1.9	1.1	0.42
Imaging diagnosis	0.4	0.3	0.7	0.7	0.8	0.6	0.7	0.5	0.7	0.6	0.56
Functional tests	0.3	0.5	0.1	0.2	0.1	0.2	0.2	0.4	0.1	0.4	0.90
Procedures	1.2	1.3	1.8	2	2.8	2.6	3.4	2.9	2.8	2.6	0.28
Medication	2.7	1.6	8.1	5.7	11.5	8.3	12.4	11.5	10.7	9.7	0.07
Equipment and supplies	0.1	0.1	0.2	0.2	0.4	0.4	0.4	0.3	0.3	0.3	0.03
Total cost	6.4	0.4	14.1	8.6	19.6	11.0	20.9	14.9	18.3	12.9	0.03
Duration of hospitalization (days)	5.3	1.5	8.4	4.7	9.3	2.8	9.3	4.4	8.9	4.1	0.26
Hospitalization cost per day	1.3	0.3	2.8	4.5	2.1	1.1	2.7	3.4	2.5	3.2	0.35

* GOLD A, B, C, D: the Global Initiative for Chronic Obstructive Lung Disease (GOLD) Classification A, B, C, D.

**Table 3:** Associated factors with AECOPD hospitalization cost.

Characteristics	Coef.	SE	*p*-Value	95% CI	
Disease stages (vs GOLD A)					
GOLD B	1.15	2.67	0.67	−4.09	6.39
GOLD C	4.10	3.69	0.27	−3.13	11.32
GOLD D	5.78	2.58	0.03	0.73	10.83
Age	0.37	0.12	0.00	0.13	0.61
Sex (female vs male)	−4.24	5.74	0.46	−15.49	7.01
Accommodation (urban vs rural)	2.29	2.69	0.40	−2.99	7.57
Smoking status (vs current smoker)					
Former smoker	−4.12	3.83	0.28	−11.63	3.39
Never smoke	−5.89	5.98	0.33	−17.62	5.83
Duration of hospitalization	1.91	0.32	0.00	1.28	2.53
Number of times hospitalized due to AECOPD in the last 12 months	0.11	0.68	0.88	−1.24	1.45
Having comorbidities (yes vs no)	1.00	2.41	0.68	−3.73	5.73
Comorbidities	−3.60	2.25	0.11	−8.00	0.81
Hypertension (yes vs no)					
Diabetes Mellitus (yes vs no)	−1.96	3.54	0.58	−8.90	4.99
Congestive heart failure (yes vs no)	−3.61	4.32	0.40	−12.07	4.85
Coronary artery diseases (yes vs no)	−1.68	3.32	0.61	−8.19	4.83
Cor pulmonale (yes vs no)	3.06	2.45	0.21	−1.75	7.86
GERD (yes vs no)	−0.14	3.19	0.97	−6.40	6.12
Constant	−22.73	12.27	0.06	−46.78	1.31

AECOPD: Acute exacerbations of chronic obstructive pulmonary disease; GERD: Gastroesophageal reflux disease.
